# Feasibility of Endoscopic Lumbar Discectomy in a Remote Government Hospital in Thailand: A Cost-Utility Analysis

**DOI:** 10.7759/cureus.52673

**Published:** 2024-01-21

**Authors:** Nantaka Tepaamorndej, Thitikan Wangapakul, Ambar Elizabeth Riley Moguel, Abdel Raouf Kayssi, Niimron Nisahoh, Saowakhon Artasar

**Affiliations:** 1 Neurosurgery, Yala Regional Hospital, Yala, THA; 2 Neurosurgery, Institute of Security and Social Services of State Workers, Mexico City, MEX; 3 Neurosurgery, Arkansas Neuroscience Institute, Arkansas, USA; 4 Community Nursing Health Care, Yala Regional Hospital, Yala, THA

**Keywords:** endoscopic, government hospital, rural areas, microdiscectomy, cost-utility, endo-discectomy

## Abstract

Background: Treatments for lumbar discectomy have developed over time. Recently, endoscopy has played an important role. However, a major obstacle to endoscopy in rural areas is the cost of surgery, particularly for endoscopes and disposable equipment. We assessed the cost effectiveness of endoscopic lumbar discectomy compared to the traditional open microdiscectomy technique in a government hospital in a developing country.

Methods: This study focused on 50 patients who underwent endoscopic lumbar discectomy between April 2019 and March 2020 at Yala Regional Hospital and were reviewed by our team. The duration of hospital stays, operative time, follow-up, and clinical outcomes at one, three, and six months postoperatively were observed and compared with 30 patients who underwent microscopic lumbar discectomy. Hospital expenses were calculated and compared using t-tests.

Results: Endoscopic discectomy was 4.00 days length of stay while microscopic discectomy has 9.77 days in averages. The pain score was 8.82 for endoscopic surgery and 9.1 for microscopic surgery. The operative price for the endoscopic discectomy was 144.69 USD higher than that for the open lumbar discectomy because of the disposable equipment. However, each patient in the microdiscectomy group had a longer hospital stay and required more perioperative care, which decreased the difference of the total hospital expenses (1,420.612 vs 1,399.16 USD).

Conclusion: Full endoscopic lumbar discectomy is an effective procedure that is beneficial for patients. The total hospital costs are not significantly different between the two procedures. To ensure that more patients receive this benefit and to develop surgical competency in government hospitals, the surgical reimbursement fee for endoscopic discectomy should be more affordable than that for conventional discectomy.

## Introduction

Lumbar disc herniation is a common degenerative disease in patients presenting with lower back pain. In the 1930s, Mixter and Barr developed a discectomy for lumbar disc herniation [1]. Open lumbar discectomy is the standard procedure. In 1997, Foley and Smith developed an endoscopic discectomy technique (transmuscular approach) for lumbar disc herniation, which allowed spine surgeons to reliably decompress symptomatic lumbar nerve roots via an endoscopic and minimally invasive surgical approach [2]. In 2006, Ruetten et al. introduced a full endoscopic interlaminar discectomy technique using an optimal system and newly developed instruments for lumbar degenerative diseases [3,4].

An endoscopic technique was developed to minimize the secondary iatrogenic instability associated with open surgery and dural scarring. More satisfactory clinical results were obtained by applying the full endoscopic technique compared with conventional open discectomy [5]. Full endoscopic discectomy provides significant benefits to patients compared to conventional open surgery in terms of hospitalization, postoperative pain, wound complications, and a more rapid return to the workplace; therefore, its use is spreading worldwide [6-10]. However, endoscopic equipment such as cameras, drills, and bipolar devices incur initial costs. Furthermore, endoscopes and surgical instruments are small and vulnerable, increasing equipment maintenance costs. This may be why endoscopic spine surgery is still not applicable in regional hospitals in low-income countries.

Since we are in rural areas where most of patients cannot afford any extra cost, we conducted a retrospective analysis using medical data to assess the total cost during hospital stays, operating costs, net profits, and hospital loss between endoscopic lumbar discectomy and conventional microscopic discectomy. One of the most important obstacles to novel treatments such as endoscopic spinal surgery is cost effectiveness. In this study, we attempt to determine the exact problems and identify possible solutions. 

## Materials and methods

Study design and patient population

In this retrospective descriptive comparative study, a total of 50 patients who underwent endoscopic lumbar discectomy, either interlaminar endoscopic lumbar discectomy (IELD) or transforaminal endoscopic lumbar discectomy (TELD), from April 2019 to March 2020 in Yala Regional Hospital were enrolled. The inclusion criteria were patients who had typical radicular pain, and MRI revealed disc herniation at any lumbar level correlated with clinical findings. Exclusion criteria were previous surgery at the same level of the lumbar spine; severe spinal stenosis; segmental instability; other pathological conditions such as acute inflammation, infection, fractures, or tumors; patients who needed to convert from an endoscopic to a microscopic procedure intraoperatively; and loss of follow-up within six months.

A consent form was signed before the surgery and the processing of manuscript was approved by ethical committees of the hospital. The operation was performed using two techniques, namely, the interlaminar or transforaminal approach, depending on the MRI findings and the surgeon’s discretion. Follow-up examinations were performed one, three, and six months postoperatively. Postoperative MRI was performed in some patients with suspected residual or recurrent disc herniation.

Data collection

Demographic and baseline data included age, sex, clinical presentation (pain, numbness, claudication, and/or weakness), symptom duration, lumbar level of disc herniation, MRI findings (disc protrusion, extrusion, or sequestration), length of hospital stay, and operative time. The patients’ clinical outcomes included the visual analog scale (VAS) score for pain after the operation and during the follow-up period. The hospital costs for each patient were collected for hospital statement analysis.

We compared our data with the collection of microdiscectomy cases, and data were obtained from 30 cases we performed in the previous years before switching all our cases to endoscopic procedures. Patients from rural areas tended to seek treatment only after the disease had advanced. Microdiscectomy causes muscle injury and bone defects, so we preferred posterior fusion and instrumentation. Pure microdiscectomy was reserved only for those who had isolated disc herniation. When performing endo-discectomy, we could remove the disc and decompress directly from the neuron canal with less bone destruction, so we tended to perform it in those who had borderline disease. Operative time, duration of hospital stays, procedure costs, and total hospital expenses were calculated and compared.

Statistical analysis

Statistical analysis Continuous variables are expressed as means. Categorical variables are expressed as numbers and percentages. The Student’s t-test was used to compare continuous variables, and the chi-square test was used for categorical variables, as appropriate. All statistical analyses were 2-tailed, and statistical significance was set at P < 0.05. Data were analyzed using PASW Statistics 18 (SPSS Inc., Chicago, USA).

Surgical technique

All surgeries were performed with the patient in the prone position on a radiolucent frame under general or local anesthesia. The interlaminar endoscopic approach was performed under general anesthesia, whereas the transforaminal approach was performed under local anesthesia. Intraoperative fluoroscopy was performed to ensure the correct positioning of the endoscope. Successful removal of the herniated disc was determined using intraoperative findings (dural pulsation, loose neural elements, retrieved disc fragments, intraoperative symptoms, and postoperative symptoms) and postoperative MRI in some cases.

Interlaminar Approach

The patients were placed in the prone position with their hips flexed to increase the interlaminar space. A 9 mm skin incision was made under fluoroscopic guidance as close to the medial side in the craniocaudal middle of the interlaminar window as possible. A 7 mm dilator was bluntly inserted into the lateral edge of the interlaminar window under fluoroscopic guidance to confirm its position. Subsequently, an 8 mm diameter operative sheath with a beveled opening was directed toward the ligamentum flavum. An endoscopic camera was used, and the procedure was performed under direct visual control and constant normal saline irrigation. A lateral incision window of approximately 4-6 mm was made in the ligamentum flavum. The neural structures and epidural fat tissues were exposed. An operating sheath with a beveled opening can be turned and used as a nerve hook. The joystick principle, medial and lateral and cranial and caudal mobility within the spinal canal, can be used to search for and remove the protruding disc using disc-punch forceps. In some cases, with narrow interlaminar spaces, we enlarged them with a laminectomy rongeur and drill.

Transforaminal Approach

The patients were placed in the prone position with their hips flexed with minimal intravenous sedation. Preoperative imaging and intraoperative fluoroscopy were performed to establish the entry site. The skin entry angle depends on the patient and is generally 8-15 cm lateral to the midline [11]. The approach angle for the disc depends on its direction and location. The skin was locally anesthetized using 1% lidocaine. A localization needle was advanced to the Kambin’s triangle under fluoroscopic guidance in two planes, anterior-posterior and lateral view. A guide pin was inserted through the puncture needle into the intervertebral disc, and the obturator and cannula were inserted after performing an 8-10 mm skin incision. Subsequently, dilation and endoscopic exploration were performed. The endoscope was docked into the targeted disc space using Kambin’s triangle. The herniated disc was removed using the inside-out technique. To corroborate the complete removal of the herniated disc, the cannula was moved towards the epidural space, and pulsation of the dural sac was observed, which confirmed the indication for decompression [12].

## Results

50 patients who underwent endoscopic lumbar discectomy between April 2019 and March 2020 at Yala Regional Hospital were enrolled. Patient characteristics are summarized in Table 1. The mean patient age was 40 (ranging from 19 to 65), which showed no specific different between two groups (p value = 0.446). 40 and 10 patients underwent IELD and TELD, respectively. The operated levels included L3-4 (four patients, 8%), L4-5 (28 patients, 56%), and L5-S1 (18 patients, 36%). All 50 patients presented with pain, numbness (37 patients, 74%), weakness in the extremities (18 patients, 36%), and neurogenic claudication (seven patients, 14%). The most common type of disc herniation on MRI was disc protrusion (25 patients, 50%), followed by disc extrusion (14 patients, 28%) and disc sequestration (11 patients, 22%). The demographic and clinical characteristics of the microdiscectomy patients were not statistically different (p value > 0.05).

**Table 1:** Demographic and clinical characteristics of patients. This table shows the demographic and clinical characteristic of patients who underwent surgery both endoscopic surgery (IELD or TELD) and microscopic surgery. IELD: Interlaminar endoscopic lumbar discectomy; TELD: Transforaminal endoscopic lumbar discectomy

Clinical characteristics	Endoscopic surgery			Microscopic surgery	P-value
	IELD	TELD	Total		
Number of patients	40	10	50	30	
Age (years)	19-65	19-54	19-65	19-68	
Age average (years)	39.60	38.80	39.4	40.20	.446
Sex					
Male	20	6	26	20	.147
Female	20	4	24	10	
Level					
L3-4	1	3	4	11	.764
L4-5	26	5	28	42	
L5-S1	13	2	18	26	
Symptoms					
Pain	40	10	50	30	-
Numbness	29	8	37	26	.180
Weakness	13	3	16	15	.110
Claudication	4	3	7	N/A	
Mean duration of symptoms (months)	7.05 (1-24)	5 (2-12)	6.64 (1-24)	6.53 (1-24)	.710
Preoperative MRI					
Disc protrusion	18	7	25	N/A	N/A
Disc extrusion	11	3	14	N/A	
Disc sequestration	11	0	11	N/A	

Clinical outcomes

Clinical outcomes are summarized in Table 2. The total length of stay for endo-discectomy was four days, whereas for microdiscectomy, it was 9.77 days. Significantly longer length stay was observed in the microdiscectomy group (p value < 0.001). This length of the hospital stay was affected by cultures and educational status of patients in rural areas which required more time to learned how to take self-care after surgery. There were no differences in length of hospital stay between IELD and TELD, which were 4.08 and 3.6 days, respectively.

**Table 2:** Clinical outcomes after treatment. This table shows clinical outcomes after treatment of patients who underwent endoscopic discectomy (IELD and TELD) and microscopic discectomy. VAS: Visual analog scale; IELD: Interlaminar endoscopic lumbar discectomy; TELD: Transforaminal endoscopic lumbar discectomy

Clinical outcome	Endoscopic surgery			Microscopic surgery	P value
	IELD	TELD	Total		
Mean length of hospital stays (days)	4.1	3.60	4.00	9.77	<0.001
Mean operative time (hours)	2.82	1.9	2.640	3.44	<0.001
Pain VAS (0-10)					
Preoperative	8.77	9	8.82	9.1	.515
Postoperative 1 day	2.55	3	2.64	2.5	.824
Postoperative 1 month	0.63	0.8	0.66	1.17	.294

**Figure 1:**
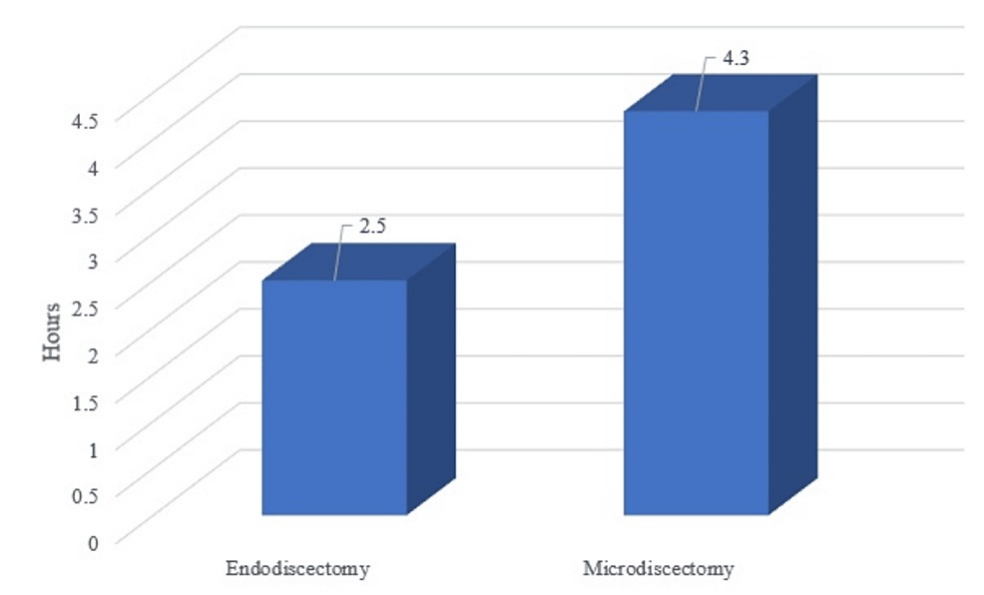
Mean operative time. This graph compares mean operative time between endoscopic and microscopic lumbar discectomy. The endoscopic procedures take significantly less time than microscopic procedures.

The mean operative time for endo-discectomy (Figure 1) was 2.64 hours and 3.44 hours for microdiscectomy, demonstrating that endo-discectomy required a significantly shorter time (p value < 0.001). TELD has a shorter operative time than IELD because it is performed under local anesthesia and requires less bone work and soft tissue management.

**Figure 2:**
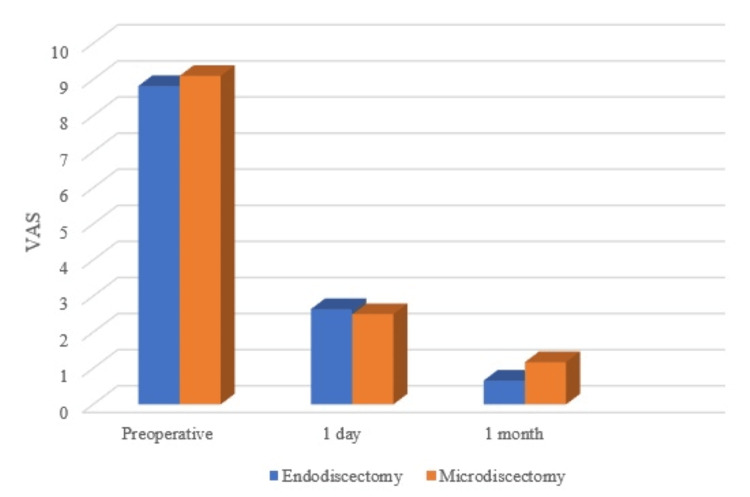
VAS for pain between endoscopic and microscopic lumbar discectomy. This graph shows VAS for pre-operation, one day after surgery, and one month after surgery. Microscopic discectomy showed a higher score but was not statistically different. VAS: Visual analogue scale

Overall, the VAS score for back and leg pain (Figure 2) showed significant improvement immediately and one month postoperatively in every treatment modality without statistical difference.

Hospital statement

**Figure 3:**
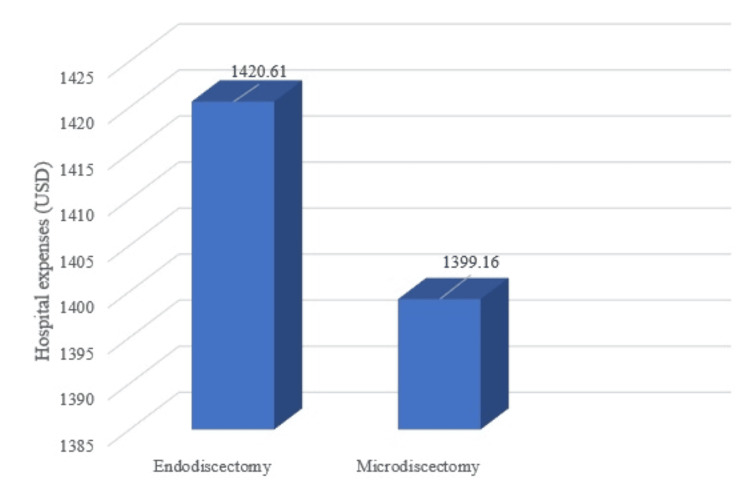
Total expenses of each procedure. This graph shows that the total expense of endoscopic discectomy is higher than microscopic discectomy.

Overall hospital statement is shown in Table 3. The average hospital expense for endoscopic discectomy was 1,420.61 USD/patient and 1,399.16 USD/patient for microdiscectomy (Figure3). This showed statistically different (p value = 0.007). There was a difference in IELD (1,431.20 USD) and TELD (1,378.27 USD) because the anesthetic cost was higher in IELD. The hospital income for each case was calculated by adjusting the relative weight (RW), which in most cases was 3.54. The hospital cost for microscopic discectomy was 870.25 USD and was fully paid for by the government. The surgical code specified by the Thailand National Health Security Office (NHSO) was 08.51, the adjusted RW for hospital stay was 3.54, 1 RW was 245.83 USD, and the diagnosis, including surgical procedure reimbursement fee per case was 870.25 USD. The same payment from the government was used for endoscopic procedures. In total, the government paid 870.25 USD per case for endoscopic discectomy; the hospital income was lower than the expense, with an average negative amount of 550.36 USD/patient for the endoscopic and 528.91 USD/patient for the microscopic procedure.

**Table 3:** Comparison of hospital statements in each modality of treatment. This table shows details of hospital statements for patients who underwent endoscopic discectomy (IELD and TELD) and microscopic discectomy. Endoscopic discectomy cost more than microscopic discectomy (p value = 0.007). RW: Relative weight; IELD: Interlaminar endoscopic lumbar discectomy;  TELD: Transforaminal endoscopic lumbar discectomy

Hospital statement	Endodiscectomy			Microdiscectomy	P value
	IELD	TELD	All		
Average hospital expense (USD/patient)	1,431.20	1378.27	1,420.612	1,399.16	0.007
Adjust RW	3.54	3.54	3.54	3.54	
Hospital income (USD/patient)	870.25	870.25	870.25	870.25	
Hospital loss (USD/patient)	560.95	508.02	550.3616	528.91	

The initial cost for a basic endoscopic system, including an endoscope at 0- and 30-degree, xenon light source HD camera, full HD screen with HD recording, high-speed drill system, irrigation system, multi-radio frequency system, and assistive devices, was 167,000 USD. Disposable devices include needle guides and tubing sets, disposable lines, bipolar probes, and shaver blades for high-speed drills. For endoscopic procedures, the total disposable equipment price for 50 cases was 7,235.39 USD, the disposable equipment per case was 144.69 USD, the average hospital expense per case was 1,420.61 USD, and the average hospital loss per case was 550.35 USD (Table 4).

**Table 4:** Equipment prices for 50 cases. This table compares the equipment prices for patients who underwent IELD and TELD. IELD: Interlaminar endoscopic lumbar discectomy; TELD: Transforaminal endoscopic lumbar discectomy

	Unit price (USD)	Used quantities	IELD	TELD	Total (USD)
Endoscope	167,000				
Needle guide	47.5	10	Not use	Use	475
Tubing set	113	3	Use	Use	339
Bipolar probe	773	4	Use	Use	3,092
High-speed drill blade	832	4	Use	Not use	3,328

## Discussion

Based on the results of the surgery in our study, we conclude that after mastering the endoscopic procedures, endoscopic lumbar discectomy has more advantages than microdiscectomy because it requires a shorter hospital stay and less operative time. The VAS score was not significantly different; according to several meta-analyses, micro- and endo-discectomy did not show significantly different outcomes for this parameter [13].

Several publications have studied cost effectiveness. Choi et al. stated that endoscopic discectomy is more cost-effective than microscopic discectomy at one year [14]. Golan et al. compared societal factors [15]. Wang et al. reported a higher expense for IELD than for TELD, but no statistical differences were observed [16]. In developing countries, especially in government hospitals and rural areas, endoscopic equipment costs remain a major obstacle. Hospital expenses for microdiscectomy seem to be lower because all equipment is reusable and can be used for other operations, not only spinal procedures. Kim et al. performed a cost-utility analysis of endoscopic discectomy in Korea, which was appropriate for quality-adjusted life years [17]. Qu et al. showed a lower price for microscopic discectomy, but endoscopy is still worth performing because of the lower invasiveness of the procedure [18]. However, higher expenses for perioperative care make the difference in total hospital expenses between these two procedures smaller. Overall, the negative values of the hospital statements were not significant.

Hospital income per case was calculated from the diagnosis-related group (DRG) and adjusted RW, which mainly depended on the principal diagnosis, operative procedure, and hospital stay. In Thailand, the surgical code for endoscopy has not yet been clarified. Until now, it remains the same as that for open microdiscectomy. Thus, disposable endoscopic equipment costs cannot be charged for by the NHSO. The option Yala Regional Hospital uses to reduce hospital costs is the reuse of disposable equipment. Disposable equipment is no longer used when it is broken. The average reuse was 10-12 times for each piece of equipment. In some cases, damage occurs during preparation, cleaning, and sterilization; therefore, the hospital executive should settle for specific personnel responsible for this fragile equipment. Another option to reduce costs is to increase the surgical reimbursement fees from the government. As suggested by a study from a developed country, higher fees should be set depending on the complexity of the procedure and the benefits to the patient [19]. Nevertheless, even with somewhat increased surgical fees, cost-effectiveness owing to the widespread availability of endoscopic surgery will have a greater impact on national healthcare cost savings.

Limitations of this study 

Expenses of this case were converted from Thai baht to USD, which may cause slight different in value. These expenses vary from hospital to hospital. Price of instruments are different for eevery country as well as government's budget for the surgery. Length of hospital stay may be affected by other factors such as patient's concern. 

## Conclusions

Full endoscopic lumbar discectomy is a novel effective procedure with benefits to patients. It provides less invasive, less than half length of hospital stay compared to microdiscectomy, and better long-term post operative pain improvement after one month. Although the procedure costs more than microscopic surgery, the overall expenses which comprise of surgical expenses, hospital expenses are not statistically different. To provide greater benefits of endoscopic surgery to patients and develop surgical competency in government hospitals, the surgical reimbursement fee for endoscopic discectomy should cover all expenses. There is a great possibility for endoscopic discectomy to be done widely in general hospitals in the future.
